# Pitfalls of whole exome-sequencing: hidden *DYNC2H1* mutations in patients with Jeune asphyxiating thoracic dystrophy

**DOI:** 10.1186/2046-2530-1-S1-P80

**Published:** 2012-11-16

**Authors:** H Arts, M Schmidts, EMHF Bongers, MM Oud, LEM Duijkers, Z Yap, J Stalker, JL Yntema, A Hoischen, C Gilissen, JA Veltman, A Kutkowska-Kaźmierczak, EJ Kamsteeg, PJ Scambler, PL Beales, NVAM Knoers, R Roepman, HM Mitchison

**Affiliations:** 1 Department of Genetics, Radboud University Nijmegen Medical Centre, the Netherlands; 2 Molecular Genetics Molecular Medicine Unit, UCL Institute of Child Health, UK; 3 UK10K, Wellcome Trust Genome Center, Cambridge, UK; 4 Department of Medical Genetics, Institute of Mother and Child, Poland; 5 Department of Medical Genetics, University Medical Centre Utrecht, the Netherlands

## 

In recent years whole-exome sequencing has been developed, a technique by which all exons of the genome (all the protein-coding DNA) can be sequenced at once. Here we show that whole-exome sequencing, using either 35 or 50 Mb Agilent kits for exome capture, was insufficient to detect pathogenic DYNC2H1 variants in patients with Jeune asphyxiating thoracic dystrophy (ATD; MIM208500). Jeune syndrome is a rare inherited ciliopathy involving chondrodysplasia characterized by shortened ribs and long bones, and polydactyly, progressive kidney and liver disease as well as retinitis pigmentosa. Reduced thoracic capacity causes approximately 60% early lethality. DYNC2H1 encodes a subunit of the dynein 1B motor that drives tip-to-base ciliary intraflagellar transport, and mutations have previously been associated both with embryonically lethal short rib-polydactyly and the milder, but overlapping Jeune aspyxiating thoracic dystrophy. Although the DYNC2H1 gene was targeted in our whole-exome experiments many sequence reads were not properly aligned, resulting in 30-70% of the gene not being covered. Only a combination of whole-exome sequencing and a candidate gene approach (i.e. analysis of non-covered DYNC2H1 exons using Sanger sequencing) enabled us to detect the missing DYNC2H1 mutations. Whole-exome data analysis of the 90 exon DYNC2H1 gene is therefore comparable to playing ‘hide and seek’, whereby certain mutations are easier to find than others according to their relative coverage. In conclusion, although whole-exome sequencing has revolutionized the field of human genetics, our findings emphasize that next-generation sequencing also presents significant challenges for gene identification and for implementation of this technique in DNA diagnostics.

